# Quality of life and factors affecting it in patients with Alzheimer’s disease: a cross-sectional study

**DOI:** 10.1186/s12955-020-01554-2

**Published:** 2020-09-10

**Authors:** Burcu Akpınar Söylemez, Özlem Küçükgüçlü, Merve Aliye Akyol, Ahmet Turan Işık

**Affiliations:** 1 Department of Internal Medicine Nursing, Dokuz Eylul University Faculty of Nursing, Inciralti, Izmir, Turkey; 2 Department of Geriatrics, Dokuz Eylül University, Faculty of Medicine, İzmir, Turkey

**Keywords:** Alzheimer’s disease, Quality of life, Caregivers, Proxy

## Abstract

**Background:**

Quality of life (QoL) is a growing area of interest in dementia research. However, it remains a controversial topic. This study aimed to determine the QoL of people with Alzheimer’s disease (PwAD) and investigate the factors affecting patients’ and caregivers’ QoL scores.

**Methods:**

A cross-sectional study design was used. A total of 98 home-dwelling PwADs and their primary caregivers were recruited in the study. Sociodemographic characteristics and QoL scores, activities of daily living (ADL) and instrumental ADL (IADL), Mini-mental State Examination (MMSE) scores, neuropsychiatric inventory (NPI), and NPI–distress were determined to assess the relevant outcomes. All statistical analyses were performed using SPSS version 22.0. Descriptive statistics, t-test, Pearson correlation, and multinomial regression were used for analysis.

**Results:**

The patients’ ratings of their QoL were higher than those of the caregivers. Caregiver education, patients’ ADL, and IADL were associated with the patients’ score on the Quality of Life in Alzheimer’s Disease (QoL-AD) scale. In addition to these variables, MMSE, NPI, and NPI–distress were associated with the caregiver scores on QoL-AD.

**Conclusion:**

From a clinical point of view, the proxy-rated scores of QoL cannot replace the self-ratings of the patients. This study suggests that both self- and proxy-rated QoL scores should be applied whenever possible. Focusing on the management of behavioral problems and supporting functionality and cognitive functions may be modifiable factors that may represent targets for intervention to improve the QoL. The findings of this study should also be used to design caregiver educational programs about the determinants of QoL.

## Background

Alzheimer’s disease (AD), the most common type of dementia, is an important health problem in aging populations [1, 2]. Although the quality of life (QoL) is a growing area of interest in dementia research, it remains a controversial topic. The QoL of people with AD (PwAD) is considered an individual, subjective, dynamic, multidimensional, and complex construct and includes the assessment of and adaptation to the consequences of AD [3].

Measuring the QoL of PwAD is a controversial issue given that the disease progresses with cognitive impairment. Several researchers have reported that PwAD have limited quantification of QoL due to their cognitive impairment and noncognitive symptoms, such as depression and psychosis [4, 5]. On the other hand, patients should be the main source of information about their own lives, and evidence indicates that PwAD can also comment on their own QoL [6–9]. For this reason, researchers are searching for ways to measure the QoL meaningfully and accurately in PwAD. A recent review has identified nine QoL measures for people with dementia and has assessed their psychometric properties [10]. Most measures were based on proxy assessment, with questionable validity for people with mild to moderate dementia. The best researched measure was the Quality of Life in Alzheimer’s Disease (QoL-AD) scale [9].

This scale is available as a self-rating version [QoL-AD Self-Rating (QoL-AD-SR) scale] and as a proxy rating version (QoL-AD Proxy Rating (QoL-AD-PR) scale). The instrument has been translated in various languages, and the Turkish version has good psychometric properties overall [11].

Discrepancies exist between patients and caregivers regarding the QoL of PwAD, and factors have been associated with divergent ratings [12, 13]. Not only the patients’ but also the relatives’ and professionals’ perceptions of the patients’ QoL should be considered [14]. The factors associated with the QoL-AD by self- and proxy ratings have been reported in many recent studies [15–20]; however, none of them have used a Turkish sample. One meta-analysis pointed out that future work should explore whether PwAD in lower-income countries or other cultures can identify different factors [21]. Testing a subjective concept, such as QoL-AD, as well as the factors associated with QoL-AD in different cultures and in different sociodemographic characteristics will contribute toward shaping an appropriate care based on culture. Researchers should consider that QoL-AD can have cultural differences. In addition to this, improving the QoL is an important focus of various therapeutic interventions as an outcome measure to evaluate interventions for Alzheimer’s care [22].

Therefore, this study aimed to investigate the mutual relationships among cognitive impairment, behavioral symptoms, functional abilities, and QoL as evaluated in PwAD and their proxies as well as to identify the factors affecting self’ and proxies’ QoL ratings.

## Methods

### Participants

A cross-sectional and correlational design was used. Sample selection was conducted using nonprobability convenience sampling. A total of 98 dyads of persons with mild to moderate AD who were all home-dwelling as well as their primary caregivers were recruited in this study. The National Institute of Neurological and Communicative Disorders and Stroke and the Alzheimer’s Disease and Related Disorders’ Association criteria were used for the diagnosis of probable AD [23]. The inclusion criteria were as follows: a family member of the patient and the primary person responsible for caregiving and providing care for at least 6 months. The exclusion criteria for the caregivers were those with visual, hearing, or speech impairments. PwAD could be taking cholinesterase inhibitors, memantine and cholinesterase inhibitors, or memantine alone and have a Mini-mental State Examination (MMSE) score of 10 and higher. Patients with severe dementia; those with psychiatric disorders, such as severe depression, schizophrenia, and bipolar disorder; those with other neurological diseases and other types of dementia (such as vascular dementia, multi-infarct dementia, or Lewy body dementia); and uncontrolled clinical problems, such as hypertension, diabetes, were excluded. In the current study, because the PwADs with MMSE score of 10 and higher could usually complete the scale without problems, patients with severe dementia were excluded. The power of the study was evaluated using G. Power 3.1. For QoL-AD-PR, a sample size of 98, effect size of 0.44, and alpha value of 0.05 were considered, and the power of study was found to be 0.99. For QoL-AD-SR, a sample size of 98, effect size of 0.18, alpha value of 0.05 were considered, and the power of study was found to be 0.82. Generally, a power of 0.80 is acceptable for such studies.

### Questionnaires

#### Participant information form

The form was prepared to obtain the sociodemographic information (such as gender, age, years of education, caregiving period, and relationship) of PwAD and their caregivers. It was filled by caregivers.

#### QoL-AD

This scale is available in two parts: QoL-AD-SR and QoL-AD-PR. QoL-AD-SR was completed by patients and QoL-AD-PR was completed by caregivers. The scale contains 13 items concerning physical health, energy, mood, living situation, memory, family, marriage, friends, the individual as a whole, ability to perform chores, ability to conduct recreation activities for fun, money, and life as a whole. The 13 domains are rated as poor (1), fair (2), good (3), or excellent (4), and the total score ranges from 13 to 52 [9]. A high score indicates good QoL. The questions of the scale are simple, clear, and understandable for easy understanding of PwAD who have cognitive failure [9]. The scores are calculated separately for self-reported and proxy ratings. The total score is calculated by multiplying the score obtained through the answers of patients by two, adding the score of caregivers, and then dividing the answer by three [9]. Cronbach’s alpha values for the self-reported and proxy ratings for the Turkish population were in the high range (0.84 and 0.77, respectively) [11].

### CDR scale

This scale was used to determine the possible stages of cognition and function: 0 (no dementia), 0.5 (questionable dementia), 1 (mild dementia), 2 (moderate dementia), and 3 (severe dementia) [24]. It was determined by the physician (fourth researcher) in the research team.

#### MMSE

MMSE comprises items regarding orientation, learning, short-term memory, language use, comprehension, and basic motor skills. It is used to assess the cognitive function. The total score ranges from 0 to 30. A low score indicates high cognitive impairment [25].

### ADL

The ability of PwAD to perform functional activities was assessed with Barthel ADL and Lawton’s IADL [26, 27].

#### NPI

NPI was used to investigate the presence and severity of neuropsychiatric symptoms; the scores range from 0 to 144, with high scores corresponding to severe behavioral disorders [28].

#### NPI–distress

The NPI has a distress section to evaluate caregiver distress toward behavioral symptoms. The distress of care was assessed as perceived by the caregivers of PwAD. The total score ranges from 0 to 60. A high score indicates a high level of perceived distress.

### Procedures

The study was conducted between June 2017 and July 2018 at a dementia outpatient clinic in the west part of Turkey. The data were collected during follow up of the patients at the outpatient clinic. The administration of the questionnaire lasted for 25 min. Caregivers filled the scale by themselves considering the QoL-AD they care for. PwAD responded to questions about their QoL by verbally.

Approval to conduct the study was obtained from the ethical committee of the Dokuz Eylul University, Noninvasive Research Ethics Board (2017/21–45). The participants who agreed to participate in the study (after written permission had been obtained) were asked to complete the questionnaires. PwAD and their caregivers had a face-to-face contact with the researcher.

### Statistical analyses

All statistical analyses were performed using SPSS version 22.0. The Shapiro–Wilk test was used to verify the normal distribution of variables. Parametric variables were described by their mean and standard deviation (SD) and nonparametric variables were described by their median and interquartile ranges.

For the QoL-AD scores, the average of the self-reported and proxy scores were compared using the student’s t-test. Correlational analyses (Pearson’s r) were performed to clarify the relationships among cognitive impairment, behavioral symptoms, functional abilities, caregiver distress, and QoL. The stepwise forward multiple linear regression analysis was performed to identify the optimal model of predictors that explain the outcome variable of interest. The “enter” method, in which all the variables are included in the model, was used. The variance inflation factor (VIF) and tolerance were used to detect multicollinearity between the independent variables in the regression model. The independent variables with VIF > 10 were removed from the model, and tolerance was less than 0.20. For all analyses, the level of statistical significance was set at *p* ≤ 0.05.

## Results

A total of 98 home-dwelling patients were included. The mean ages of patients and caregivers were 73.58 (SD = 9.37) and 56.85 years (SD = 13.64), respectively. A total of 65 patients (66.3%) and 58 caregivers (59.2%) were women. Among the caregivers, 52 (53.1%) were the children of the patients and 46 (46.9%) were spouses.

The mean MMSE score was 17.38 ± 4.31, and 68.4% of patients had mild AD. The mean NPI and NPI–distress scores were 22.86 ± 18.82 and 11.85 ± 9.52, respectively. Table 1 presents the sociodemographic and clinical characteristics of the study population.

**Table 1:** Sociodemographic and clinical characteristics of participants

	Mean	SD
**Patient characteristics**		
Gender Female (%)	66.3	
CDR (%): 1 / 2	68.4/31.6	
Age	73.58	9.37
Years of education	8.03	4.54
MMSE	17.38	4.31
NPI	22.86	18.82
ADL	91.02	15.43
IADL	4.59	2.69
NPI-Distress	11.85	9.52
QoL-AD-SR	38.30	4.26
**Caregiver characteristics**		
Gender Female (%)	59.2	
Age	56.85	13.64
Years of education	11.75	4.91
Caregiving period	3.03	2.41
Relationship with the patient		
Child (%)	53.1	
Spouse (%)	46.9	
QoL-AD-PR	31.58	4.85

*Abbreviations*: *ADL* Activities of Daily Living, *IADL* Instrumental ADL, *MMSE* Mini-mental State Examination, *NPI* Neuropsychiatric Inventory, *QoL-AD* Quality of Life in Alzheimer’s Disease, *QoL-AD-SR* QoL-AD Self-Rating, *QoL-AD-PR* QoL-AD Proxy Rating, *CDR* Clinical Dementia Rating

Comparisons of the total QoL-AD scores between patients (38.30 ± 4.26) and caregivers (31.58 ± 4.85) revealed a statistically significant difference (t = 64.40, *p* < 0.001). The patients rated a higher QoL than the caregiver group.

No statistically significant difference was observed between the QoL assessments of patients who are cared for by spouses and children (t = 0.801, *p* = 0.090).

Self-reported QoL score was the only sociodemographic variable that showed correlation with caregiver education (*p* = 0.001). No significant differences were observed in age, gender, and care duration. No significant differences were noted the in self-reported QoL scores across different CDR stages, whereas caregivers reported poor QoL in the moderate stage (Table 2).

**Table 2:** Correlation of the QoL-AD-SR and QoL-AD-PR with study variables

Variable	Self-score		Proxy-score	
	QOL-AD		QOL-AD	
	r	***p***	r	***p***
QOL-AD-SR	1.00	.001	.377	.001
QOL-AD-PR	.377	.001	1.00	.001
Caregiver education	.33	.001	.237	.019
ADL	.25	.012	.238	.018
IADL	.235	.020	.443	.001
MMSE	.165	.104	.400	.001
NPI	-.007	.949	-.532	.001
NPI-Distress	-.057	.576	-.571	.001

*Abbreviations*: *ADL* Activities of Daily Living, *IADL* Instrumental ADL, *MMSE* Mini-mental State Examination, *NPI* Neuropsychiatric Inventory, *QoL-AD* Quality of Life in Alzheimer’s Disease, *QoL-AD-SR* QoL-AD Self-Rating, *QoL-AD-PR* QoL-AD Proxy Rating

Pearson correlation coefficient revealed that PwAD self-rated QoL showed a positive relationship with IADL (*p* = 0.020) and ADL (*p* = 0.012). Pearson correlation coefficient also revealed that the proxy-rated QoL of the patients showed a positive relationship with IADL (*p* < 0.001), ADL (*p* = 0.023), and MMSE (*p* < 0.001). A negative relationship was observed between NPI total score (*p* < 0.001) and NPI–distress (*p* < 0.001) and the proxy-rated QoL (Table 2).

This study also aimed to determine the most important predicting variables that explain the largest proportion of variance of self- and proxy-rated QoL. The detailed results are shown in Table 3. Analysis revealed that caregivers’ education years, ADL, and IADL explained 12% of the variance in self-rated QoL, whereas caregivers’ education years, ADL, IADL, MMSE, NPI, and NPI–distress explained 26% of the variance in proxy-rated QoL (Table 3).

**Table 3:** Regression models of factors predicting self-rated Qol, proxy-rated QoL

	Beta coefficient	R^**2**^	Adjusted R^**2**^	***P*** value
**Self-rated QoL**				
Caregiver education	.333 (*p* = .001)			
ADL	.252 (*p* = .006)	.149	.122	<.010
IADL	.235 (*p* = .001)			
**Proxy-rated QoL**				
Caregiver education	.237 (*p* = .009)			
MMSE	.400 (*p* = .001)	.306	.260	<.010
ADL	.238 (*p* = .009)			
IADL	.443 (*p* = .001)			
NPI	-.402 (*p* = .001)			
NPI-Distress	-.404 (*p* = .001)			

Note: Non-significant variables for self-rated QoL: MMSE, NPI, NPI-Distress

*Abbreviations*: *ADL* Activities of Daily Living, *IADL* Instrumental ADL, *MMSE* Mini Mental State Examination, *NPI* Neuropsychiatric Inventory, *QoL-AD* Quality of Life in Alzheimer’s Disease, *QoL-AD-SR* QoL-AD Self-Rating, *QoL-AD-PR* QoL-AD Proxy Rating

## Discussion

In this cross-sectional and prospective study, the caregivers’ education, ADL, and IADL of the PwAD were associated with the QoL-AD-SR. Additionally, MMSE, NPI, and NPI–distress were associated with the QoL-AD-PR scores.

Our analysis showed no differences in terms of gender of the informal caregiver, as stated by Römhild et al. [17], Schumann et al. [15] and Martyr [18]. However, this result was different from the findings of Conde-Sala et al. [13]. The objective of the study by Conde-Sala et al. (2014) was to analyze burden and mental health in the caregiver subgroups according to gender and relationship to the patient (husbands, wives, sons, and daughters). It could be possible that previous relationships with the patient can affect this result.

In the current study, the relationship with informal caregiver showed no influence on PwAD, similar to the findings of Huang et al. [29] and Römhild et al. [17]. Unlike the findings of Robertson et al. [16], ve Schumann et al. [15] concluded that the average QoL-AD score of the caregivers as the spouse of patients were higher than that of the children [30, 31]. The lack of difference in our study was attributed to culture and the commitment of the children toward their parents. This result might be due to strong family bonds and the sensibility of caregiving for the PwAD at home in the Turkish society.

According to the results of this study, self- and proxy-rated QoL are not related to age. This result is similar to that of Römhild et al. [17]. In a recent systematic review and correlational meta-analysis by Martyr et al. [18], age was not associated with the QoL. However, Barbe et al. [20] and Schumann et al. [15] have stated that age has an inverse relationship with the QoL. In a study conducted by Barbe et al. [20], the mean age of patient was 82 years (it was 73 years in the current study). This could have had an effect on the lower QoL. In consequence of using the cohort method in Schumann et al. [15], the results can be different with the current study.

### Differences between self and proxy QoL of AD

Patients rated a significantly higher QoL than the caregivers, which is in accordance with the results of previous studies [12, 13, 15–20]. As stated by other researchers, this situation is thought to be due to patients’ insufficient insight, awareness of caregivers about their patients’ condition, and negative outcomes such as burden of care. Disease severity affects caregivers more than the patients themselves [32]. In addition, patients having reduced abilities to judge their own difficulties due to increased cognitive impairment may affect this situation [12]. In this regard, caregivers may assume that the QoL of the patient with impaired cognition also worsens.

### Factors affecting QoL-AD

#### Factors affecting QoL-AD-SR

According to the results of this research, caregiver education and the functional level of patients affect their self-reported QoL. Unlike this study result, Römhild et al. [17] and Martyr et al. [18] concluded that caregiver education is not related to QoL. Different from the findings in literature, in this study, education in providing care is an important variable in the care of patients and may have affected the QoL positively. Given that educated caregivers are conscious about the approach and care of their patients, this positive approach is thought to enable patients and caregivers to achieve positive QoL.

The ratings of both QoL scores are low if the patient is functionally impaired. Functionality, as stated by Schumann et al. [15], Römhild et al. [17], and Martyr et al. [18], is related to QoL. High self-reported QoL scores indicate an excellent functional status. Functionality is one of the indispensable components of life. The current relation result is logical because the need for functional support generally stems from the presence of multiple functional impairments, and the patient’s perception of their own health is heavily influenced by the presence of such functional impairments.

#### Factors affecting QoL-AD-PR

Caregiver education years, functionality, cognition, behavioral symptoms, and distress affect proxy-rated QoL. Similar to the finding of this study, Sousa et al. [33] stated that caregiver QoL ratings are influenced by the caregivers’ educational level [33]. Educated caregivers who are aware of their patients’ condition are thought to have a positive attitude toward the patients’ QoL.

High QoL scores are an expected outcome for patients with excellent functional state. It was observed that individuals with high functionality have a high QoL score [18]. A caregiver who sees their loved one suffering from several functional impairments is highly normally expected to rate the patient’s QoL more negatively.

In this study, significant correlations were observed between proxy-rated QoL and worsened cognitive impairment. Self-rated patient QoL showed association with the said factor. However, QoL is a controversial topic. The findings of the current study are inconsistent with those of the literature. Conde-Sala et al. [31] stated that no significant correlation exists between patient- and caregiver-reported QoL and cognitive function. The results of this study on cognitive function are similar to those of other studies [12, 13, 18].

Caregivers experience difficulty in managing behavioral symptoms. High behavioral symptoms negatively affect proxy-rated QoL. A strong negative relationship exists between behavioral symptoms and proxy-rated QoL [15]. Hongisto et al. [19] examined the effect of NPI on the QoL of PwAD longitudinally over a five-year period. Although the behavioral problems of the patients increased in the five-year period, self-rated QoL showed no change; however, proxy-rated QoL decreased gradually against the increasing behavioral symptoms observed by the caregivers.

In this study, significant correlations were observed between proxy-rated QoL and caregiver distress. The results of this study are similar to those in the literature [15, 17, 18, 20]. Using regression analysis, Fuh et al. [34] observed that caregiver distress (NPI–distress) was among the determinants affecting proxy-rated QoL.

### Regression analysis

Overall, our fitted regression models accounted for 12 and 26% of the observed variance of the difference between self-reported and proxy ratings. Therefore, other unknown factors influence the difference between self and proxy ratings, and they were not addressed by the data of our analysis.

## Conclusion

AD has become a health concern due to its dramatically increasing prevalence. This condition may result in serious consequences for patients, their caregivers, and health services. For these reasons, the QoL of PwAD and the factors affecting patients’ and caregivers’ QoL rating must be determined. Our study highlights the statistically significant different caregiver- and self-rated QoLs. This study showed that the discrepancy between patient- and caregiver-reported QoL showed no change based on cultural boundaries. Low self- and proxy-rated QoL ratings were observed with low caregiver education and functionally impaired patients. Proxy-rated QoL was also rated low if the patient had poor cognitive status and behavioral problems as well as if the caregivers had suffered from behavioral problems related to distress.

From a clinical point of view, the proxy-rated QoL rating cannot replace the self-rating of PwAD. This study suggests that both self- and proxy-rated QoL ratings should be applied whenever possible. Focusing on the management of behavioral problems and supporting functionality and cognitive functions may be modifiable factors that may represent targets for intervention to improve the QoL. The findings of this study should be used to design caregiver educational programs about the determinants of QoL.

Several limitations have to be considered in our study. First, the findings are specific to patients with mild to moderate AD and who live in their homes. Therefore, the results cannot be generalized to people with severe AD or institutionalized patients. In addition, the emotional aspects of caregivers, such as depression and anxiety, were not evaluated. Future research may focus on QoL changes over time. The QoL of late-stage PwAD and the factors that affect patients’ and caregivers’ QoL rating must also be determined.

## Data Availability

The datasets used and/or analyzed during the current study are available from the corresponding author on reasonable request by burcu.akpinar@deu.edu.tr

## Abbreviations

AD: Alzheimer’s Disease
QoL: Quality of Life
PwAD: People with Alzheimer’s Disease
ADL: Activities of Daily Living
IADL: Instrumental ADL
MMSE: Mini Mental State Examination
NPI: Neuropsychiatric Inventory
QoL-AD: Quality of Life in Alzheimer’s Disease
QoL-AD-SR: QoL-AD Self-Rating
QoL-AD-PR: QoL-AD Proxy Rating
CDR: Clinical Dementia Rating
